# Laparoscopic Distal Pancreatectomy for Pediatric Blunt Pancreatic Injury: A Case Series

**DOI:** 10.7759/cureus.92440

**Published:** 2025-09-16

**Authors:** Maho Kurashima, Hannah Wiseman, Shin Miyata, Justin A Sobrino

**Affiliations:** 1 Pediatric Surgery, Cardinal Glennon Children’s Hospital, St. Louis, USA; 2 Pediatric Surgery, Saint Louis University School of Medicine, St. Louis, USA; 3 Pediatric Surgery, Children’s Healthcare of Atlanta, Atlanta, USA

**Keywords:** case series, distal pancreatectomy, minimally invasive surgery, pediatric surgery, traumatic pancreatic injury

## Abstract

Pancreatic injuries in pediatric patients are rare and present challenges in diagnosis and treatment due to patient size and limited evidence-based guidelines. This case series highlights the laparoscopic management, decision-making process, and outcomes of three pediatric patients with grade III pancreatic injuries.

Case 1 involved a two-year-old male with a near-complete transection of the pancreatic tail following a motor vehicle collision. He underwent laparoscopic distal pancreatectomy 24 hours post-injury, complicated by ileus and mild pancreatitis, which resolved without intervention. Case 2 was another two-year-old male with a complete pancreatic transection from blunt trauma, who underwent laparoscopic distal pancreatectomy 36 hours post-injury and was discharged uneventfully. Case 3 was a 12-year-old male with a mid-body pancreatic transection after a handlebar accident, who underwent delayed laparoscopic distal pancreatectomy seven days post-injury following failed pancreatic duct stenting. Postoperatively, he developed pancreatic leakage and bowel perforation, requiring additional surgeries, including a colostomy and further pancreatic resection.

Laparoscopy was selected in all cases due to hemodynamic stability and enhanced visualization of the pancreatic duct and splenic vessels. In Cases 1 and 2, small patient size precluded endoscopic retrograde cholangiopancreatography, prompting early surgical intervention. In Case 3, delayed surgery after failed stenting led to inflammation and significant postoperative complications. This series emphasizes the importance of timely surgical decision-making in pediatric pancreatic injuries. While laparoscopy offers advantages, challenges such as pancreatic duct identification and the risk of conversion to open surgery require careful intraoperative assessment.

## Introduction

Blunt trauma to the abdomen is a significant cause of abdominal injuries in children. Among these, pancreatic injury ranks as the fourth most common solid organ injury, occurring in 0.3-9.5% of cases, following injuries to the spleen, liver, and kidney. Morbidity and mortality rates range from 26.5-60% and 5.3-30%, respectively [1-3]. Accurate diagnosis of pancreatic injury and its severity is crucial for determining an appropriate treatment strategy. Although CT is considered the best initial imaging modality for blunt abdominal trauma, its sensitivity and specificity are reduced in pediatric patients. In adults, endoscopic retrograde cholangiopancreatography (ERCP) is a highly specific and reliable method for diagnosing pancreatic ductal injury; however, its use, along with magnetic resonance cholangiopancreatography (MRCP), is controversial in pediatric trauma settings and may not always be feasible due to patient size.

The management of blunt pancreatic trauma in pediatric patients remains a topic of debate. Treatment typically depends on the grade of pancreatic injury, particularly whether the pancreatic duct is involved [4,5]. Non-operative management is preferred for grade I and II injuries [3]. However, there are no established guidelines for escalating to operative management in grade III or higher injuries, nor is there a consensus on whether open or laparoscopic approaches are more effective. Open management is traditionally regarded as the standard of care for pancreatic surgeries, but it carries higher risks, including larger incision sites, increased infection rates, and prolonged hospitalization [6]. Laparoscopic management offers several advantages, such as improved visualization of the pancreatic duct and splenic vasculature, smaller incisions, faster recovery of bowel function, and shorter hospital stays [7,8]. Nonetheless, laparoscopic approaches in acute trauma settings pose challenges, including longer operative times, limited exploration of associated injuries, and technical difficulties in smaller patients [7,9].

To date, only 25 cases of laparoscopic distal pancreatectomy for blunt abdominal trauma in the pediatric population have been reported since 2001. Here, we discuss three cases of pediatric patients who sustained traumatic pancreatic injuries necessitating laparoscopic distal pancreatectomy. This manuscript was prepared in accordance with the CARE guidelines.

## Case presentation

Case 1

**Figure 1:**
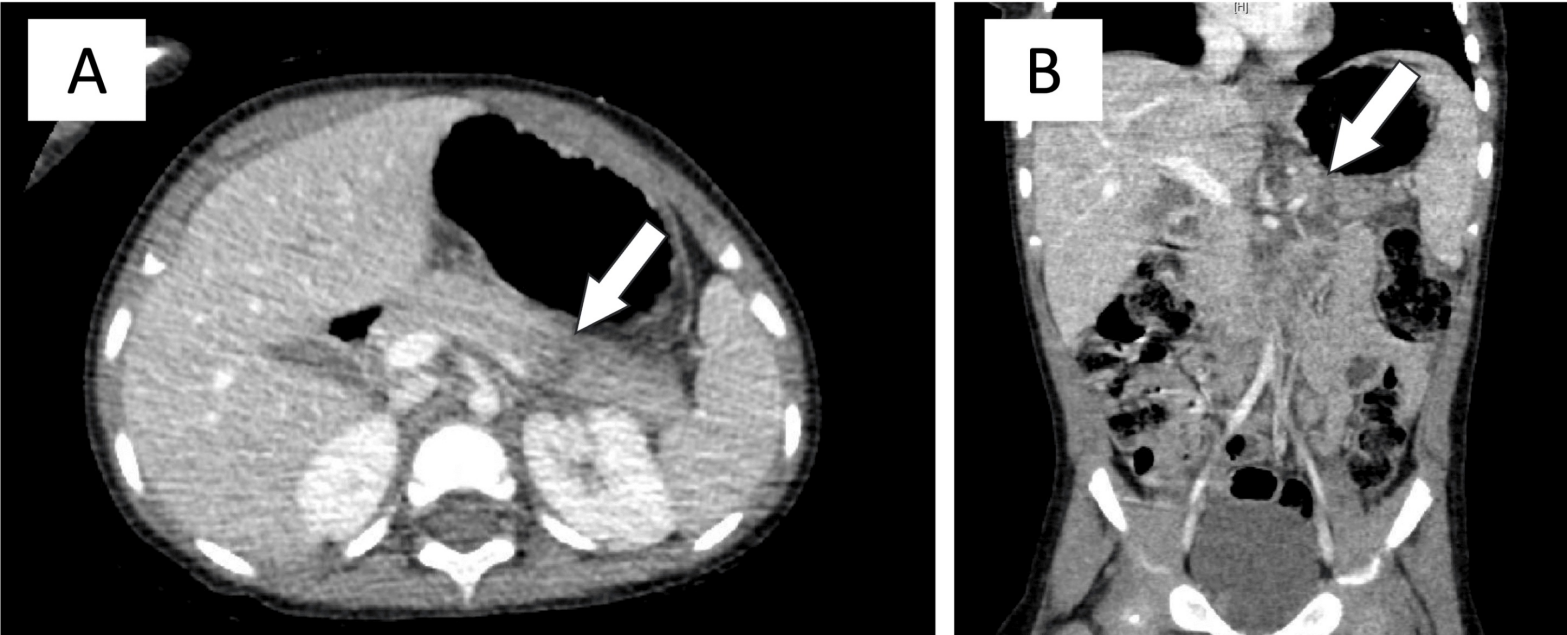
Case 1: Abdominal CT with contrast. (A) Axial view. Transverse lucency (white arrow) through the distal body and tail of the pancreas with adjacent edema in the pararenal space, extending toward the inferior spleen. A grade III left kidney injury is also observed. (B) Coronal view. Transverse lucency (white arrow) can be observed. Findings were compatible with a grade III pancreatic injury.

The patient was a two-year-old male involved in a motor vehicle collision (MVC) as an unrestrained backseat passenger. The car was found rolled over and crashed into two trees. Three fatalities in the vehicle were reported. The patient was found awake underneath the front seat of the car. On arrival at the hospital, the patient exhibited normal vital signs on room air except for mild tachycardia of 135 beats/minute. Physical examination exhibited a non-tender, non-distended abdomen without guarding or bruising. Laboratory findings revealed a normal complete blood count (CBC), including hemoglobin (Hb) of 12 g/dL, and an elevated lipase level of 530 U/L. Mild hematuria was noticed on urinary analysis. All other laboratory findings were within normal limits. Abdominal contrast CT scan revealed a transverse laceration of the distal body and tail of the pancreas with adjacent fluid inferolaterally, as well as a grade III left kidney injury (Figures 1A, 1B). Due to the patient’s size, ERCP and MRCP were not feasible for investigating pancreatic ductal injuries. Following admission, 24 hours after the initial injury, the patient experienced progressive abdominal pain and tenderness. A decision was made to proceed with diagnostic laparoscopy and laparoscopic distal pancreatectomy.

**Figure 2:**
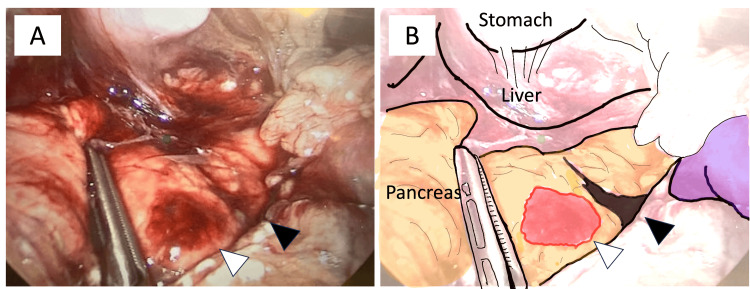
Case 1: Diagnostic laparoscopy findings. (A) Transverse laceration of the distal body and tail of the pancreas with adjacent fluid accumulation inferolaterally. The white arrowhead indicates a parenchymal hematoma, while the black arrowhead denotes a complete transection of the parenchyma. (B) Illustration of the intraoperative findings showing a near-complete transection of the pancreas.

**Figure 3:**
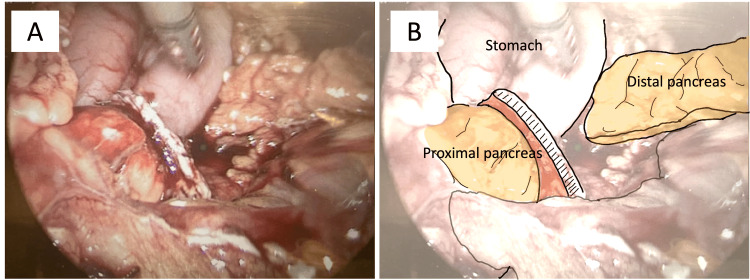
Case 1: Intraoperative laparoscopic image illustrating a distal pancreatectomy using a stapler. (A, B) The distal pancreas was divided using an endoscopic stapler. The pancreatic duct was completely sealed with staples.

**Figure 4:**
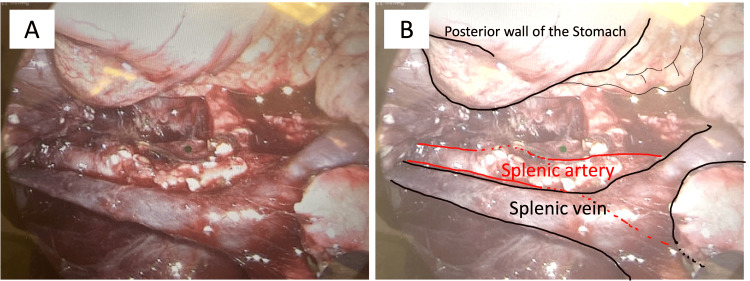
Case 1: Intraoperative laparoscopic image showing splenic vasculature preservation. (A, B) The splenic vein and splenic artery were isolated and preserved. Both vessels were inspected using a laparoscopic magnified view, with no evidence of vascular injury.

A 10 mm trocar was inserted in the umbilicus. Two additional 5 mm trocars were placed as working ports in the right flank and the right lower quadrant area. Another two 5 mm trocars were placed in the left flank and the epigastric area for assistant ports. The 30° angled scopes were inserted through the umbilical trocar, and a slight head-up position was achieved. Operative findings showed nearly complete resection of the pancreatic tail (Figures 2A, 2B), which was consistent with the lucency found on abdominal CT. The distal pancreas was divided using an Endoscopic stapler (Echelon 3000 45 mm Green) (Figure 3). The splenic vein and splenic artery were both isolated and preserved. There was no evidence of vascular injuries (Figure 4). An abdominal drain was placed near the staple line in the right upper quadrant. Operative time was 137 minutes with an estimated blood loss of 100 cc.

The postoperative course was complicated by ileus on postoperative day (POD) four. Total parenteral nutrition (TPN) was started. The patient had a return of bowel function with stools on POD six. The patient had a nasoduodenal feeding tube (ND tube) placed on POD seven due to persistent malnutrition and intolerance of oral feeds. The patient was able to gradually tolerate increased PO feeds, and TPN was discontinued. The ND tube was continued through POD 13. Chemical pancreatitis was noted on POD 13, with elevation of serum amylase to 238 U/L and serum lipase to 117 U/L, but resolved without intervention. The patient was discharged home on POD 14 after removal of the ND tube. The drain was removed on POD 27 in the clinic. After the most recent clinic appointment, two months postoperatively, the patient was released from further follow-up.

Case 2

The patient was a two-year-old male with no past medical history who came to the emergency department for emesis and abdominal pain that began two days prior. He reportedly fell on the playground before symptoms started; however, a clear story was not given by the family. On arrival at the hospital, the patient exhibited normal vital signs on room air except for mild tachycardia of 135 beats/minute. Physical examination revealed multiple bruises on the abdomen and back. The abdomen was non-distended and mildly tender in the upper quadrants without guarding. Laboratory findings included a normal CBC with Hb of 11.6 g/dL and elevated lipase at 270 U/L. All other laboratory findings were within normal limits.

**Figure 5:**
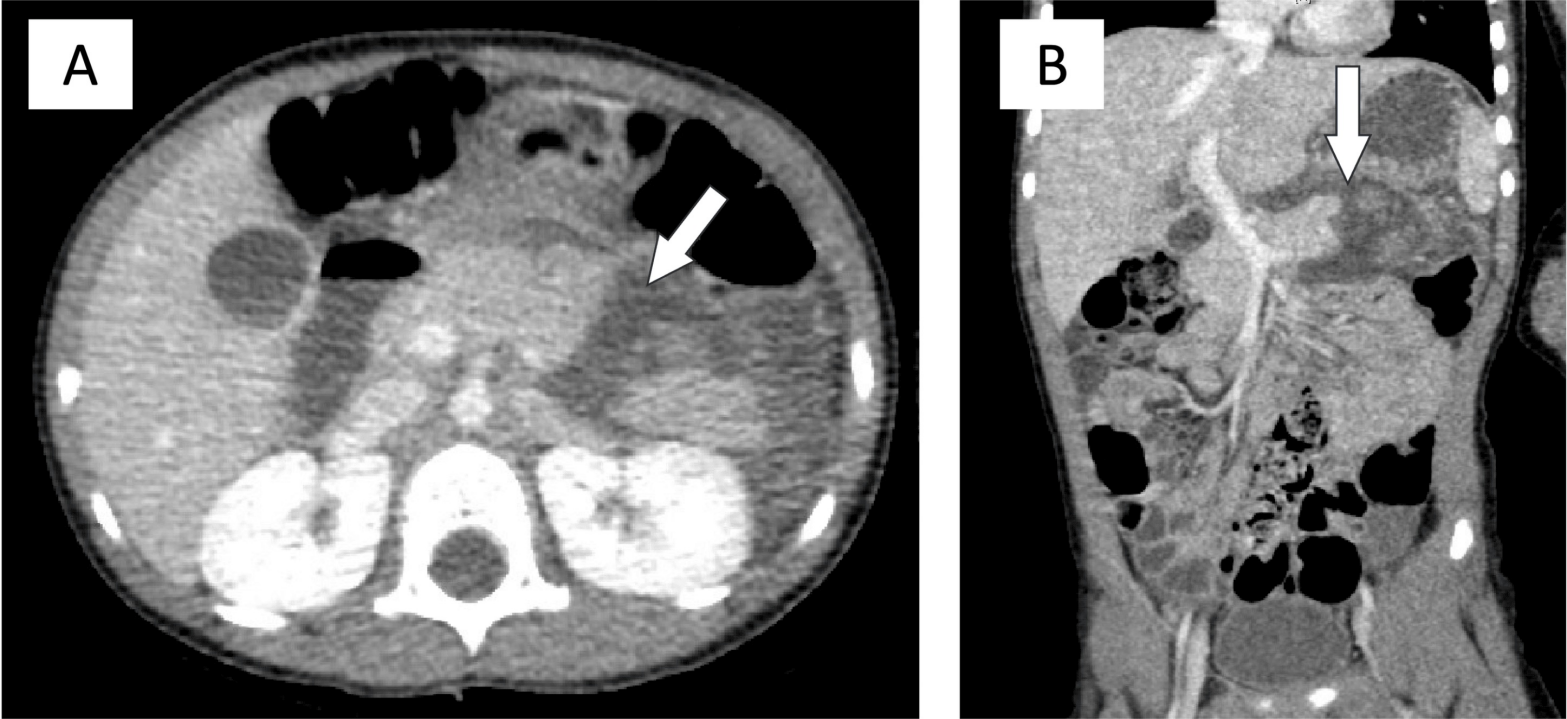
Case 2: Abdominal CT with contrast. (A) Axial view. A ductal injury and complete transection at the junction of the pancreatic body and tail (white arrow), with a thinly encapsulated fluid collection surrounding the transection. Findings were consistent with a high suspicion of a grade III pancreatic injury. (B) Coronal view.

Abdominal CT scan with contrast revealed ductal injury and complete transection at the junction of the pancreatic body and tail with a thinly encapsulated fluid collection surrounding the transection (Figure 5). This was consistent with the high suspicion of a grade III pancreatic injury. Pelvic fluid, fractures of the anterior left seventh and eighth ribs, and a buckle fracture of the anterior left ninth rib were also visualized. Due to the patient’s size, ERCP and MRCP were not feasible for investigating pancreatic ductal injuries. Following admission, 36 hours after the initial injury, the patient experienced progressive abdominal pain and tenderness. The decision was made to proceed with diagnostic laparoscopy and laparoscopic distal pancreatectomy.

Operative findings showed minimal fat saponification and a retroperitoneal hematoma. Visualization of the pancreas was achieved via retroperitoneal entry through the hematoma. The mid-body of the pancreas was revealed to be completely transected and surrounded by a mostly liquefied hematoma. The distal pancreas was resected using an Endoscopic stapler (Echelon 3000 45 mm Green). The splenic vein and splenic artery were both isolated and preserved. An abdominal drain was placed near the staple line in the right upper quadrant. Operative time was 185 minutes with an estimated bleeding amount of 50 cc.

The postoperative course was uneventful. Abdominal drain was removed on POD five, and the patient was medically ready for discharge. Non-accidental trauma, including physical abuse, was suspected based on history, physical examination, and imaging studies. The patient was matched with a foster family, secured a safe discharge plan, and discharged on POD seven.

Case 3

**Figure 6:**
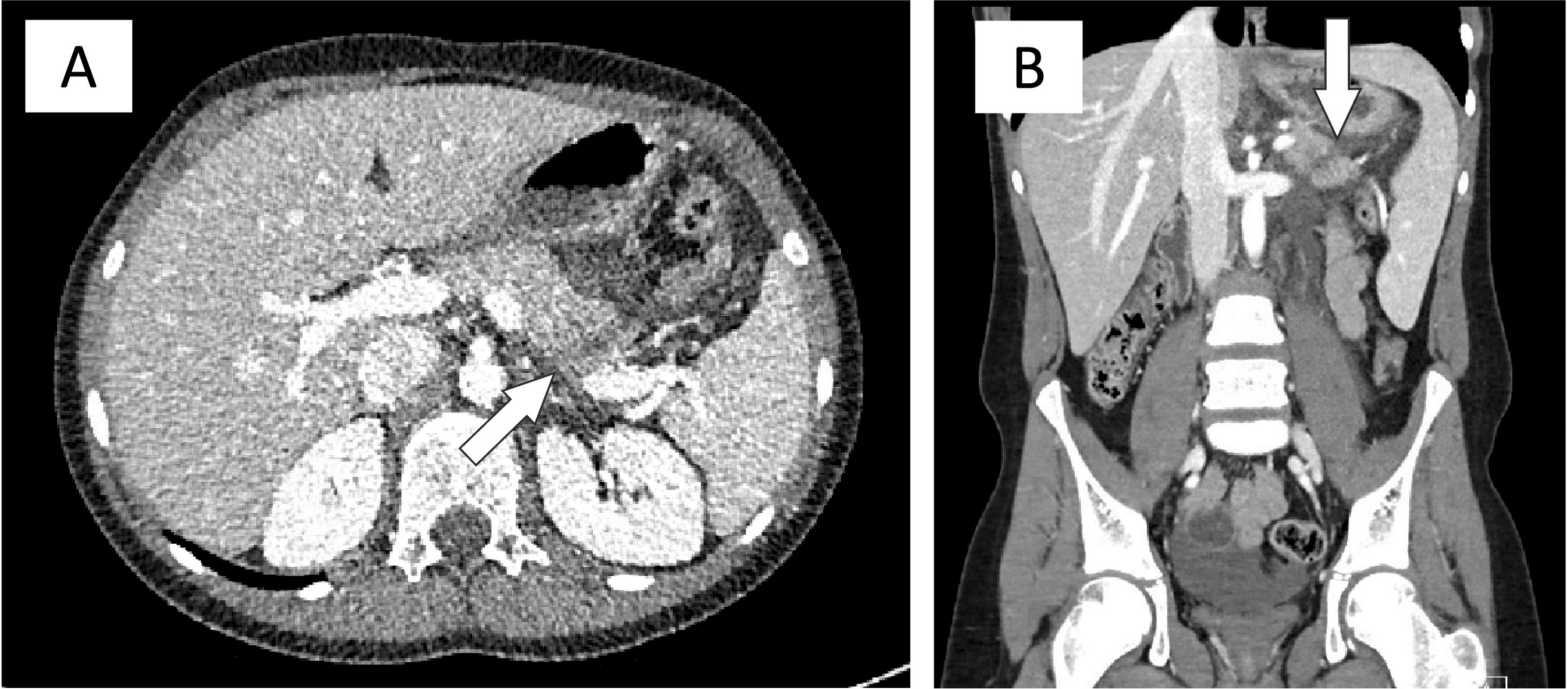
Case 3: Abdominal CT with contrast. (A) Axial view. A laceration near the junction of the pancreatic body and tail involving the majority of the pancreas (white arrow), with possible pancreatic duct involvement, consistent with a grade III pancreatic injury. Peripancreatic and retroperitoneal fluid was also visualized, extending inferiorly toward the pelvis. (B) Coronal view.

The patient was a 12-year-old male with no past medical history who sustained a handlebar injury. He presented to an outside hospital with abdominal pain and emesis two days after the initial injury. The abdomen was non-distended and tender in the bilateral upper abdomen without guarding. Laboratory findings were significant for decreased Hb of 9.2 g/L and elevated lipase at 1,254 U/L. CT demonstrated a laceration near the junction of the pancreatic body and tail across the majority of the pancreas with possible involvement of the pancreatic duct, which was compatible with a grade III pancreatic injury. There was visualization of peripancreatic and retroperitoneal fluid extending inferiorly toward the pelvis as well (Figure 6). The patient was transferred to our hospital and initially treated non-operatively with ERCP and pancreatic stent placement. The stent was unable to fully traverse the pancreatic duct transection and was instead placed approximately 2-3 cm proximal to the transection site. Endoscopic sphincterotomy was performed. The patient continued to have significant abdominal pain and worsening abdominal distention despite utilization of non-operative management. A decision was made to proceed with diagnostic laparoscopy and distal pancreatectomy on day seven following the initial injury.

Operative findings showed copious amounts of cloudy ascitic fluid and extensive inflammation and saponification at the pancreatic bed. The identification of the pancreas was extremely difficult due to inflammation and diffuse saponification. The distal pancreas was removed in a piecemeal fashion using an energy device. The transected proximal end of the pancreas was identified. Fluoroscopy was utilized to assess the distance between the tip of the pancreatic stent and the transected end. There appeared to be more than enough distance to place a stapler; however, due to the thickness of the pancreas, the stapler was unable to be closed. A 3-0 Vicryl figure-of-eight suture was placed to close the pancreatic duct with the surrounding pancreatic tissue. An additional 3-0 Vicryl figure-of-eight suture was placed to close the pancreas. FloSeal was applied to the pancreatic end. An abdominal drain was placed near the staple line in the right upper quadrant. Operative time was 185 minutes with an estimated blood loss of 50 cc.

The patient had increased abdominal distension and milky drainage through the abdominal drain as well as intermittent fevers. Repeat CT scan revealed a pelvic fluid collection, for which an abdominal drain was placed by interventional radiology on POD six. Cultures from the drain grew gram-negative rods and gram-positive cocci, which raised concern for a bowel perforation along with pancreatic leak. The patient underwent an exploratory laparotomy. A large amount of grayish fluid was drained. The bowel was severely adhered to itself, for which we liberated by performing an extensive lysis of adhesions by careful finger fracturing. There were dense adhesions to the distal transverse colon at the splenic flexure with a mesenteric injury at that level, which could have been present at the date of the trauma, as it coincided with the location of the pancreatic injury. A remnant portion of the distal pancreas was discovered as well, which was most likely the cause of the pancreatic leak. The proximal pancreas had two figure-of-eight sutures from the previous surgery, which remained in place. Distal transverse colectomy and colostomy creation, resection of remnant distal pancreas, and extensive lysis of adhesions were performed. Postoperatively, the patient remained afebrile with normal vital signs, normal WBC, and return of intestinal function; however, he still struggled with abdominal pain. The parents wanted a second opinion for surgical and medical management. The patient was transferred to another hospital. Ostomy was taken down six months later.

## Discussion

In summary, Case 1 involved a two-year-old male with a grade III pancreatic injury resulting from an MVC. Laparoscopy revealed a near-complete resection of the pancreatic tail, and a distal pancreatectomy was performed 24 hours after the injury. The postoperative course was complicated by ileus and mild chemical pancreatitis, both of which resolved without intervention. The patient was discharged on POD 14. Case 2 was a two-year-old male with a grade III pancreatic injury caused by blunt trauma. Laparoscopy revealed a liquefied hematoma surrounding the pancreas and a complete transection of the mid-body. A laparoscopic distal pancreatectomy was performed 36 hours after the injury. The postoperative course was uneventful, and the patient was discharged on POD seven. Case 3 involved a 12-year-old male with a grade III pancreatic injury following a handlebar accident. The patient underwent an ERCP; however, the stent could not traverse the pancreatic duct transection. Laparoscopic distal pancreatectomy was performed seven days after the injury. Surgery was difficult due to severe inflammation and thickened pancreatic parenchyma, which did not allow the surgical stapler to close and necessitated sutured closure of the pancreatic duct and distal pancreatic resection in a piecemeal fashion. The postoperative course was complicated by pancreatic leakage and bowel perforation, necessitating a distal transverse colectomy, colostomy creation, and resection of the remnant distal pancreas. Preoperative demographics and postoperative outcomes are summarized in Table 1 and Table 2, respectively. Cases 1 and 2 were unable to undergo ERCP due to their small size. The decision to proceed with surgery was based on clinical and CT findings suggestive of pancreatic duct injury. Although operative time and estimated blood loss were not markedly different across the three cases, intraoperative identification and ligation of the pancreatic duct and dissection of pancreatic parenchyma were significantly more challenging in Case 3. Retrospectively, the failure of pancreatic duct stenting and the delay in performing the pancreatectomy contributed to the difficulty of the surgery due to extensive adhesion and inflammation. This, in turn, resulted in the major postoperative complication of pancreatic leakage with bowel perforation.

**Table 1:** Preoperative demographics of the patients. Cases 1 and 2 were two years old and had lower body weights compared to Case 3. All cases involved blunt injuries caused by different mechanisms. The hemoglobin and lipase levels on arrival are also shown. CT scans revealed grade III injuries in all cases. Additional injuries identified on the initial CT scans are listed. Hb = hemoglobin; MVC = motor vehicle collision

Case number	Age (years)	Gender	Weight (kg)	Mechanism	Hb (g/dL, 12–16)	Lipase (U/L, 10–60)	CT grade	Other injury
1	2	Male	12	MVC	12.9	1,602	III	Left renal injury, grade III
2	2	Male	15	Abuse	11.6	279	III	Multiple rib fractures
3	12	Male	46	Handlebar injury	8.4	1,254	III	None

**Table 2:** Postsurgical outcomes. Key data include the day of surgery for laparoscopic distal pancreatectomy following trauma, details of any preoperative treatments, operative time, estimated blood loss, length of hospital stay, duration of abdominal drain placement, and complications. ERCP = endoscopic retrograde cholangiopancreatography

Case number	Surgery day from trauma	Preoperative treatment	Operative time	Operative bleeding	Length of hospital stay	Length of drain placement	Complication
1	1 day	None	137 minutes	100 cc	15 days	27 days	Chemical pancreatitis, ileus
2	3 days	None	185 minutes	50 cc	8 days	5 days	None
3	7 days	ERCP, Stent	185 minutes	50 cc	19 days	8 months	Colonic fistula, pancreatic leakage, intra-abdominal abscess

Pancreatic injury is a relatively rare occurrence in the pediatric population, accounting for approximately 0.3-9.5% of pediatric blunt abdominal traumas [1,3,8]. Ductal injury has an even lower occurrence rate with 0.12-2.9% [10]. Handlebar injuries are the most common inciting event (50%), resulting in pancreatic injury and ductal disruption [11]. Pancreatic injury can be diagnosed by elevated amylase, CT, ultrasound, MRCP, and ERCP [3,5]. While there are many diagnostic options, there are currently no specific guidelines on the steps of diagnosis and management of pediatric pancreatic injury. The status of the main pancreatic duct is a key indicator of injury severity and decision-making for whether to pursue surgical management of pancreatic injury [4,5]. Grade of pancreatic injury can be especially challenging in the pediatric population due to limited body size for ERCP or MRCP, as we experienced in Cases 1 and 2. Ultrasound has been found to have a low sensitivity and specificity when determining pancreatic injuries, with reported sensitivities ranging from 27% to 96% [11].

On initial CT, studies by Iqbal et al. and Ravindranath et al., respectively, have reported identification of transected pancreas in 75-85% of cases and identification of fluid collections in 80.5% of cases of isolated ductal injury [6,10]. Kang et al. found that the initial CT identified 13/14 pancreatic injuries in a pediatric population [12]. In contrast, CT has been reported to have a low sensitivity (38-61%) for diagnosing main pancreatic duct injuries in children [11]. Kulaylat et al. reported that one-third of pediatric pancreatic injuries were missed on initial evaluation [8]. ERCP may be a recommended and safe alternative to operative intervention and has shown improved outcomes ranging from 50% to 75% [10,13]. ERCP is the most sensitive study to confirm disruption of the pancreatic duct before surgical intervention [14]. However, there are minimal guidelines on limitations regarding the size of children. ERCP may also result in procedure-specific complications such as pancreatitis, cholangitis, duodenal perforation, and stricture formation in cases of stenting [2,4]. MRCP is a viable alternative for the diagnosis of pediatric pancreatic injuries [9,11]. A multitude of studies have recommended MRCP to be better than CT at correlating the imaging grade of pancreatic injury with management and outcome. A retrospective study from 11 pediatric trauma centers from 2010-2015 recommended that MRCP is better than CT in visualization of the pancreatic duct, but not necessarily evaluation of integrity [14]. In general, MRCP and/or ERCP are recommended when the status of the main pancreatic duct is unclear with the use of CT alone [5].

There has been an ongoing debate for many years regarding operative versus non-operative management of pancreatic injury in the pediatric population. The most common complications following pancreatic injury include formation of pancreatic fistulae, pancreatitis, pseudocysts, intra-abdominal abscess, long-term formation of ductal strictures, and development of exocrine and endocrine pancreatic insufficiency [3,8,10]. Non-operative management is the preferred management for grade I and II pancreatic injuries, but there is a clinical gray zone for the management of grade III and above [15]. Non-operative management for grade III-V pancreatic injuries has significantly higher rates of failure and complications of pseudocysts, but lower rates of reoperation compared to operative management [8,12,16]. Operative management has been associated with fewer complications, shorter time to oral feeding, and shorter length of hospital stay [2,4,6]. However, there has been no documented difference in postoperative mortality between operative management and non-operative management [6]. The downside of non-operative management would be causing delayed operative timing, resulting in failure or no improvement after several days. As we experienced in Case 3, the surgical procedure becomes difficult after developing inflammation and adhesion.

Regarding the operative approach, the laparoscopic approach has many benefits, including reduced postoperative pain, decreased intraoperative blood loss, better cosmetic results via smaller incision sites, and magnification of the operative field of view [7,17]. Laparoscopic distal pancreatectomy is associated with shorter hospitalizations and is believed to be safer as compared to open; however, there is no reported difference in morbidity and complication rates between the two [8,16,17]. It is estimated that 10.8-46.6% of published distal pancreatectomies are laparoscopic [7]. One of the biggest contraindications to the laparoscopic approach, which is especially prevalent in the trauma setting, is the decreased ability to identify injury to surrounding organs and patency of critical vasculature. The rate of laparoscopic approach to open conversion was estimated to be 0-34% [7]. The most common causes requiring conversion to open include hemorrhage and failure to progress [7]. Laparoscopic approach can also pose difficulty due to the increased technical skill required by the operating team. A multicenter analysis by Nickel et al. found no apparent learning curve-related morbidity and mortality of laparoscopic distal pancreatectomy compared with open in surgical teams with minimal laparoscopic experience [9]. A systematic review by Catellani et al. concluded that a laparoscopic approach to blunt pancreatic injury in a hemodynamically stable child is feasible and safe compared to open, specifically in the setting of an experienced laparoscopic pediatric surgical team [18]. This study did not quantify what qualifies a surgical team as experienced. When examining postoperative complications in laparoscopic versus open approach of distal pancreatectomy, the laparoscopic approach is associated with fewer postoperative complications of delayed gastric emptying and chyle leak compared to open [9]. Incidence of pancreatic fistula in laparoscopic cases varies widely at 16.8-21.7%; however, it is unclear how this rate compares to the open approach [7]. There is no reported difference in reoperation rates between laparoscopic and open approaches [7].

In our case series, operative management was chosen as a pancreatic stent was not feasible in Cases 1 and 2 due to patient size. Non-operative management via stent placement was attempted but failed in Case 3. Case 3 may have recovered faster following a definitive resection rather than with multiple failed interventions and pseudocyst formation. A laparoscopic approach was chosen for the three cases as the patients were hemodynamically stable and other organ injuries requiring surgical repair were ruled out based on preoperative CT scan. We also believed there was a potential for better assessment of the pancreatic duct injuries and splenic vessel injuries under the magnified laparoscopic vision. In the management of Case 3, it is unclear if operative management should have been pursued earlier. ERCP, when possible, is generally the recommended first step in non-operative management of pancreatic injury. However, failure of pancreatic duct stenting and delay of surgery made inflammation worse, which caused difficulty in identifying the pancreatic duct and resulted in the stapler not being applicable due to the thickness of the pancreatic parenchyma. It is also unclear whether we should have considered converting from laparoscopic to open surgery. Bowel perforation secondary to pancreatitis might have been missed intraoperatively due to limited exploration under the laparoscopic approach. Laparoscopic pancreatic duct ligation with suture might have been technically demanding and could have been the cause of incomplete ligation.

## Conclusions

We reported three cases of laparoscopic distal pancreatectomy for pediatric blunt pancreatic injuries. Our experience suggests that laparoscopy can be a feasible option, particularly for smaller pediatric patients who are not suitable candidates for pancreatic duct stenting. However, delayed operative timing may increase the technical complexity of the procedure. Given the risk of missed associated injuries and the challenges of addressing multiple injuries in a minimally invasive setting, caution is warranted when applying laparoscopic techniques in trauma. Thus, operative strategies, including the decision to transition from non-operative to operative management, as well as from laparoscopic to open surgery, should be carefully individualized.
